# Concluding Commentary: A Call for evidence in CPD and Lifelong Learning

**DOI:** 10.15694/mep.2019.000070.1

**Published:** 2019-03-26

**Authors:** Helena Filipe, Samar Aboulsoud

**Affiliations:** 1 HFAR/PL-EMGFA; 2 School of Medicine

**Keywords:** Continuing Medical Education, CME, Continuing Professional Development, CPD

## Abstract

This article was migrated. The article was marked as recommended.

The AMEE CPD Committee was invited to oversee the launch of the first themed edition of MedEdPublish on Continuing Professional Development (CPD) for 2019’s first quarter and sent a call for papers on evidence-based discussion on CPD and lifelong learning. The main aim was to highlight research and work supporting healthcare professions continuing education and professional development.

A collection of seventeen manuscripts of various types such as “New Education Method and Tools”, “Research Article”, “Case Study”, “Practical Tips or Guidelines”, “Report of Meeting or Workshop” and “Commentary” were published. The articles portray fundamental good CPD practices encompassing the array of professional competencies needed to provide high quality healthcare. The manuscripts cover various CPD subthemes among which, mentorship and feedback, interactive learning, learning communities and applied technology, interprofessional education, evaluation and assessment, research in CPD, CPD frameworks, patient safety and quality improvement. They also embrace various perspectives and cultural diversity with the deemed adjustments to the regional healthcare teams’ needs. The role and responsibilities of CPD educators are emboldened.

This edition was a platform to welcome and discuss CPD under various perspectives among the global CPD community.

## Introduction

Borderless and embracing the high diversity of regional healthcare needs and resources, Continuing Professional Development (CPD) fundamental principles and processes should entail substantial equivalency. ( Filipe, HP *et al*., 2014) While sharing many characteristics of under and postgraduate medical education across the medical education continuum, CPD presents unique challenges and opportunities. Effective CPD should be ignited by the healthcare professionals’ lifelong learning needs and CPD stakeholders (public and patients, healthcare professionals, CPD educators, employers, policy makers, regulators, professional societies) should weave collaborative leadership in facilitating continuing learning environments to advance healthcare quality. ( Aboulsoud S. and Filipe H., 2019)

**Figure 1.**
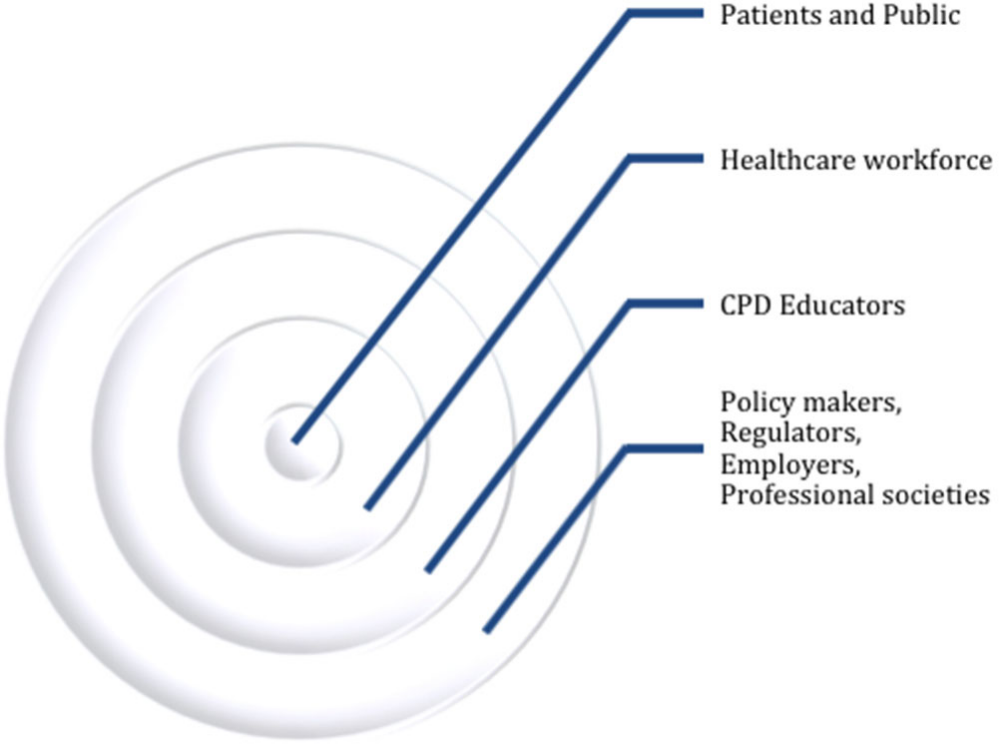
An accountable healthcare workforce performance receives guidance from patient outcomes and public health care needs. Facilitated by the CPD educator, continuing education should be supported on an instructional design with merging clinical and educational outcomes. Employers, regulators, societies, and policy makers should assume their leadership responsibility in overseeing and facilitating continuing learning environments as deemed adjusted to specific healthcare needs.

This concluding commentary closes the loop initiated with the opening editorial ( Aboulsoud S, Filipe H, 2019) and aims to provide an overview of the key issues that had emerged over the three months of a themed issue in AMEE MedEdPublish on the topic of Continuing Professional Development. We describe how this themed edition was planned and conducted, highlight the manuscripts published and suggest future direction.

## Methodology

Following on AMEE 2018 Conference and in association with the AMEE MedEdPublish Team, the CPD Committee was invited to oversee the launch of the first themed issue on CPD for 2019’s first quarter issue. The aim intended to highlight research and work done supporting and developing healthcare professions education.

Three months in advance, we wrote a call for papers on an evidence-based discussion on CPD and lifelong learning, as an opportunity for CPD professionals to present their latest research and views on current and future CPD to a wider community. Suggested sub-themes subscribe the fundamental CPD principles and a possible match of the most prominent found in each subtheme is shown in Table 1 ( Filipe *et al*., 2018 and Aboulsoud S. and Filipe H., 2018)

**Table 1.** CPD principles and subthemes proposed to authors

Principles	Subthemes suggested at the outset of the themed edition
Systematization	Accessibility and dissemination of CPD databases and resources
Comprehensiveness	Competences in CPD system
	Enhancing the utilization of health informatics and tech in CPD
	Coordination across professions for beneficial learning: proper resource utilization and effective IPE
Accreditation	CPD Accreditation
	Effective educational planning for impactful CPD
	Innovative models in CPD delivery
Regulation	CPD System/Program evaluation
Systematization, Comprehensiveness, Accreditation, Regulation ( **SCAR**)	Other topics relevant to the main theme.

The theme was promoted within AMEE networks and social media. Personal invitation letters were sent to the CPD Special Interest Group (SIG) and extended to the Global Alliance for Medical Education (GAME), the Society of Academic Continuing Medical Education (SACME), the International Academy for CPD accreditation leadership among many other CPD communities.

## Submissions and Synopsis

Fifteen manuscripts were accepted among the nineteen submitted. The seventeen articles published comprise six different manuscript types: five present a “New education method or tool, five are “Research articles”, two concentrate on “Practical tips and/or guidelines”, two present a “Case study”, one is a “Report of meeting or workshop” and two others consist on the theme guest editors” opening and concluding “Commentary”. Authors selected a variety of keywords to portray significant representations in their articles. The resultant word cloud suggests two main groups centered on the keyword “workforce”. The keywords most chosen were Development, Education, Continuing, Learning, Medical and Health followed bythe group: Excellence, Continuous, Evaluation, CPD, Social, Professions, Peer, Patient, Faculty and Training.

**Figure 2.**
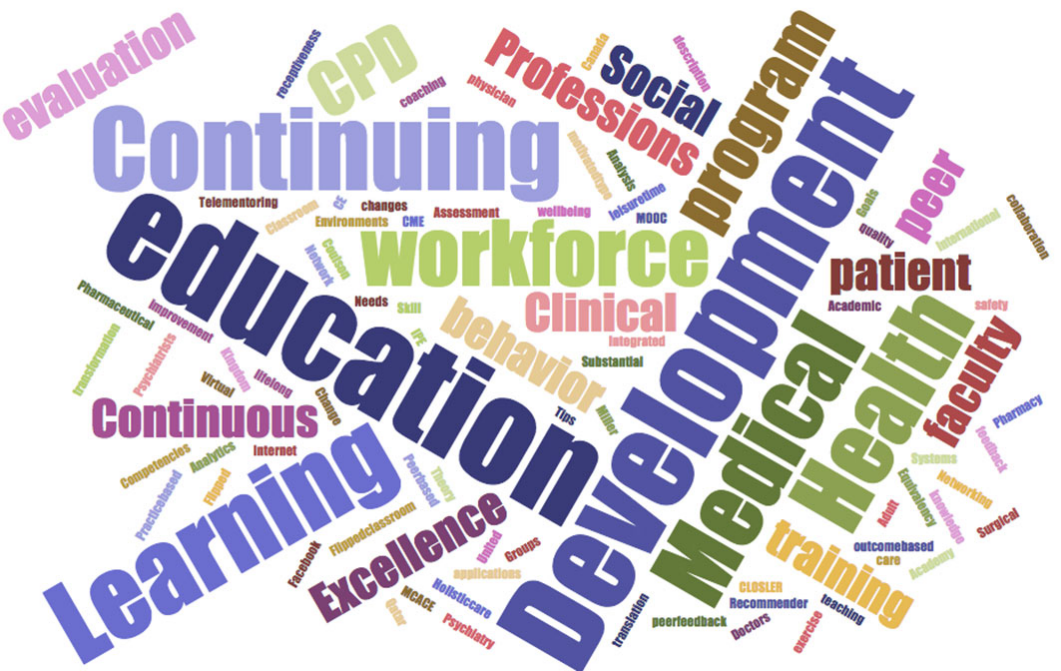
Word cloud of the manuscripts keywords.

An attempt to categorize the manuscripts within fundamental CPD principles suggested more than one per article in several of them. ( Filipe *et al*., 2018 and Aboulsoud S. and Filipe HP., 2019) The exercise highlighted the good practices to create effective CPD activities as embodied in the principle of accreditation and the holistic vision of lifelong learning needs and the broad scope of the medical profession as promulgated in the principle of comprehensiveness.

**Figure 3.**
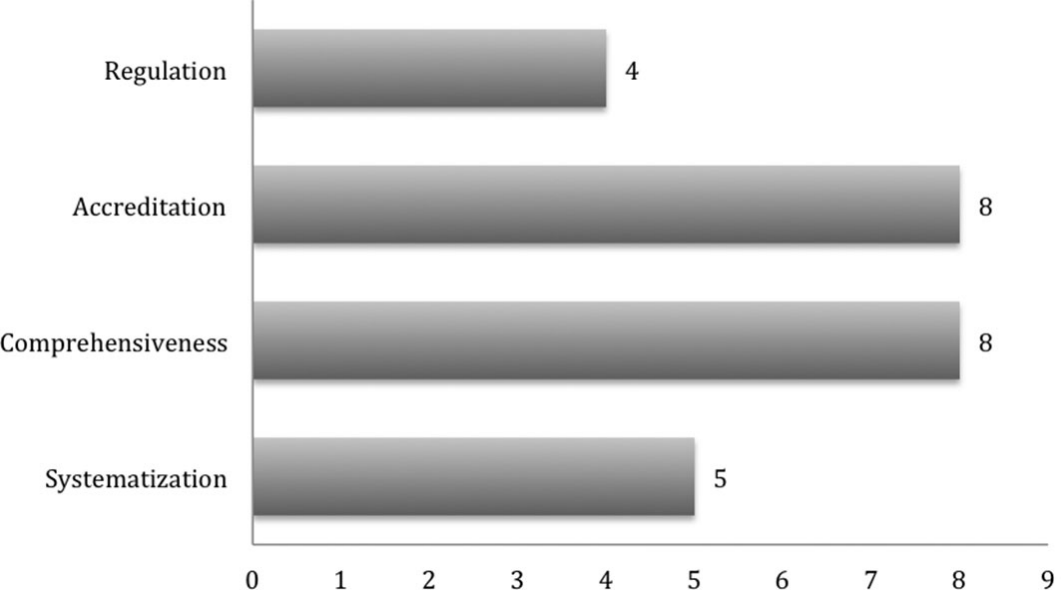
CPD principles prominently highlighted per number of manuscripts.

Table 2 presents an inventory of the manuscripts published grouped per type, their keywords and tagged prominent CPD principles. ( Filipe HP *et al*., 2018 and Aboulsoud S. and Filipe H., 2019) A brief synopsis of the manuscripts follows below.

### New Education Method and Tools

Welton and Andre designed and applied the “Best Practice Conference” (BPC) tool to use active techniques to enhance measurable learning from a continuing medical education program. They could conclude on the assessment tool’s impact on practice based learning with improvement among residents and faculty members. ( Welton R and Andre T, 2019)

Margolis and associates worked on massive open online courses (MOOCs) as “an educational opportunity to reach an international larger health professionals audience”. They “validated the new knowledge with trusted peers, in the agreement and adoption phases of change”. The intent of their work “was to create awareness in the medical education community that this type of analysis is possible and potentially useful; to receive feedback on the possible functionalities as well as critique these developments, and to create a space for collaboration in research and innovation projects with other interested parties”. ( Margolis A, López-Arredondo, García S, Rubido N,, *et al*., 2019)

Stube and colleagues developed an online community focused on clinical excellence they named “CLOSLER” aiming to be “Closer to Osler”. Conceptually supported on the professional obligation of pursuing excellence in healthcare delivery, the Miller Coulson Academy of Clinical Excellence (MCACE) (Johns Hopkins University School of Medicine) puts special emphasis in communication, interpersonal skills, professionalism and humanism, diagnostic acumen, and negotiation of the healthcare system to attain professional excellence. “The MCACE team describes Closler, a free and open access medical education website (closler.org) to share clinical stories and perspectives to stimulate reflection education that can help to move us closer to Osler and clinical excellence”. ( Stube C, Chisolm M, Miller G, Chien D, *et al*., 2019)

Koppula and Babenko developed a peer-coaching program to work as an additional source of feedback for physicians “who teach as part of their lifelong learning strategy” and are often limited to their students feedback. Despite their described investigation limitations they favor that this peer-coaching program potentially addresses the needs and barriers of physician teachers. ( Koppula S, Babenko O, 2019)

Geary and associates investigated the quality of live distance surgical mentorship as an alternative to provide CPD activities to practicing ophthalmologists. This methodology can be particularly beneficial to surgeons in remote locations where CPD activities are difficult to access. They concluded “distance surgical mentorship in phacoemulsication is an acceptable CPD training methodology for consultant ophthalmologists”. ( Geary A, Benavent S, Amador De La Cruz E, Wayman L, 2019)

### Research articles

Assuming that physicians commit to be lifelong learners with a simultaneous responsibility on their own wellbeing to deliver good care, Babenko and associates aimed to know about “(1) lifelong learning practices and leisure-time exercise habits of academic and community-based physicians; and about (2) associations of leisure-time exercise with work engagement, exhaustion, and professional life satisfaction”. They found that regardless of their practice type, physicians “tend to engage in lifelong learning activities that offer in person interactions with colleagues and trainees. Regular participation in leisure-time exercise appears to enhance physicians’ professional wellbeing. As such, these activities and habits should be encouraged, supported, and promoted within institutional culture and health systems in general”. ( Babenko O, Ding M, Koppula S, 2019)

Wu and associates suggested that training to upskill feedback giving is a feasible strategy to increase receptiveness of participants to peers’ feedback and activate participants’ motivation to make the immediate and continuous behavior changes on giving behavior-specific peer-feedback on holistic-care. ( Wu S, Yang Y, Shulruf B, Yang L, *et al*., 2019)

Yuan and associates’ study “indicated that FAIMER’s faculty development program had a positive influence in advancing several aspects in health professions continuing education. The program helped the authors to identify specific areas to strengthen and thus improving faculty development programs and its measurement. ( Yuan S, Mukherjee S, Vyas R, Burdick W, 2019)

Luconi, and associates investigated the impact of a CPD intervention focused on teaching and practicing patient safety (PS) and quality improvement (QI). Following a thematic analysis of qualitative data, descriptive statistics and triangulation of sources they could show “feasibility to develop and implement an effective CPD intervention supporting healthcare professionals’ knowledge, confidence, and reported change in teaching and practicing PS-QI”. ( Luconi F, Boillat M, Mak S, Chartrand D, *et al*., 2019)

Haruta and associates aimed to assess the validity and reliability of the Interprofessional Performance Scale in Conference (IPLSC). Their work allowed them to state that the IPLSC is a valuable evaluation tool to make consistent assessments of interprofessional performance in conferences. ( Haruta J, Yamamoto Y, Goto R, Maeno T, 2019)

### Case Study

Udo and associates investigated whether peer groups’ CPD activities within a mental health service in North East England, were compliant with guidelines. The authors recommend standards for continuous professional development. Their case study showed that to improve practice, groups should consider incorporating the approval of CPD activities and credits into meetings, setting aside time for reflection and invite external observers to comment on functioning of groups. ( Udo I, Shangobiyi A, Njoku U, Awani T, *et al*., 2019)

Bader and associates highlighted the importance of investing in the education, training and development of the global health workforce as an integral component for achieving global health goals, universal health coverage and access to safe and quality services. They advocate for a systematic and formal CPD & CE for health workers, describe a global framework model and how it is being used and implemented in countries worldwide. ( Bader L, Mukhalalati B, Awaisu A, Tofade T, *et al*., 2019)

### Practical Tips and or Guidelines

Waduud and associates with experience in the national Integrated Academic Training (IAT) pathway in the United Kingdom offer clear and useful recommendations on how to successfully apply for academic clinical training posts. ( Waduud M, Ahmed N, Scott J, 2019)

Smilski and Parrott describe and discuss two interprofessional competency frameworks already implemented in Canada and Qatar, with guidance for implementing IPE in pre- and post- licensure settings. Health care educators should follow these frameworks to plan “interprofessional education activities aimed at developing effective, collaborative practitioners”. Authors review and discuss both frameworks, which apply within their own contexts and offer opportunities for the application of interprofessional competencies globally. ( Smilski A, Parrott M, 2019)

### Report of Meeting or Workshop

Berger advances the flipped classroom combined with the workshop learning format delivery, as the methodology of choice to address the frequently missed or misinterpreted effective needs assessment piece by curriculum planners to create successful CPD activities. ( Berger G, 2019)

**Table 2.** Manuscripts published, their keywords and CPD principles prominently highlighted, grouped per manuscript type

Type of Manuscript	Title	Keywords (K) & Principles (P)
New education method or tool	The Best Practice Conference: An Interactive Practice-Based Learning Activity for Resident and Faculty Development	K: Practice-based learning; Peer-based learning; Continuing Medical Education; Flipped-classroom P: Accreditation
	Social learning in large online audiences of health professionals: Improving dialogue with automated tools	K: Continuing Medical Education; MOOC; Social Network Analysis; Learning Analytics; Recommender Systems; Virtual Learning Environments; Social Networking applications; Facebook; Internet P: Accreditation
	Getting CLOSLER (“Closer to Osler”): Developing an Online Community Focused on Clinical Excellence	K: clinical excellence; Miller Coulson Academy of Clinical Excellence (MCACE); patient care; CLOSLER; medical education P: Comprehensiveness, Systematization
	What the Teachers want - The Potential for Peer Coaching	K: faculty development; peer coaching; program description; feedback on teaching P: Regulation
	Distance Surgical Mentorship for Ophthalmologists in Northern Peru	K: Telementoring; Continuing Professional Development; Medical education; Surgical Skill Development P. Systematization, Accreditation
Research article	Lifelong learning practices and leisure-time exercise habits of academic and community-based physicians	K: lifelong learning; leisure-time exercise; physician wellbeing P: All Principles
	Engaging trainees by actively giving feedback will increase their receptiveness to peer’ feedback and motivate behavior-changes in holistic care: a pilot study	K: Holistic-care behavior; peer-feedback; motivated-type behavior changes; receptiveness P: Comprehensive, Accreditation
	Using a theory of change for evaluation: has the FAIMER international faculty development program improved the field of health professions education?	K: International Faculty Development Program; Health Professions Education; Theory of Change; Program Evaluation P: Accreditation
	Patient Safety and Quality of Care are Everybody’s Business: Evaluating the Impact of a Continuing Professional Development Program beyod Satisfaction	K: Patient safety; quality improvement; continuing professional development (CPD); knowledge translation; outcome-based evaluation; interprofessional P: Comprehensiveness, Accreditation, Systematization
	Validity Evidence for Interprofessional Performance Scale in Conference (IPSC) in Japan	K: Patient safety; quality improvement; continuing professional development (CPD); knowledge translation; outcome-based evaluation; interprofessional P: Comprehensiveness
Case Study	How Psychiatrists Continue To Learn: Operations and Practices of Continuous Professional Development Peer Groups	K: Continuous Professional Development; Peer Groups; Psychiatry; Psychiatrists; Doctors; Continuous Medical Education P: Regulation
	Using a global framework for health workforce development: National case studies on continuing professional development in pharmacy	K: Health workforce; Pharmacy; health; workforce development; continuing professional development; CPD; CE; Pharmaceutical Workforce Development Goals; workforce transformation; education & training P: Systematization, comprehensiveness, regulation, accreditation
Practical tips and/or guidelines	Twelve tips for Applying to Clinical Academic Training in the United Kingdom	K: Integrated Academic Training; United Kingdom; Tips P: Comprehensiveness
	Interprofessional Competency Frameworks in Education	K: Interprofessional collaboration; Competencies; Interprofessional education; Health education; Canada; Qatar P: Comprehensiveness, systematization
Report of meeting or workshops	Needs Assessment Lessons Learned in Qatar: A Flipped Classroom Approach	K: Continuing Health Professions Education; Flipped Classroom; Needs Assessment; Adult Learning P: Accreditation
Commentary	CPD and lifelong Learning: a call for an evidence based discussion. Opening Editorial	K: CME, CPD, Substantial Equivalency, IPE P: All Principles
	CPD and lifelong Learning: a call for an evidence based discussion. Concluding Commentary	K: Continuing Medical education, CME, Continuing Professional Development, CPD P: All Principles

## Discussion and Conclusion

This MedEdPublish themed edition on CPD has worked as a platform to promote the value of formalizing and customizing CPD systems in accordance to the regional healthcare professionals and teams needs considering the global fundamental principles of CPD good practice.

The edition provided a global venue to showcase innovative educational methods and tools, present original research, exchange case study experiences, suggest good practice guidelines, report meetings or workshops and share comments and views.

The manuscripts portray fundamental good CPD practice encompassing the array of professional competencies needed to provide quality healthcare. They cover various CPD subthemes among which, mentorship and feedback, interactive learning, learning communities and applied technology, interprofessional education, evaluation and assessment, research in CPD, CPD frameworks, patient safety and quality improvement. They embrace various perspectives and cultural diversity with the deemed adjustments to the regional healthcare teams’ needs.

This collection of manuscripts underlines the broad vision of continuing education and professional development and highlights the value of effective training pulling forward the relevant role and challenging responsibilities of CPD educators.

This edition has opened an avenue to welcome and discuss CPD, to further research useful to inform and self regulate CPD systems and programs, to encourage reflection on diverse professional perspectives and healthcare educational needs, and to showcase innovative and inspiring experiences in this breaking through area of medical education.

## Into the Future

Next April, AMEE is offering the opportunity to live discuss CPD topics during a Webinar. The ongoing CPD Webinar Series organized by the AMEE CPD Committee will continue throughout 2019, suggesting additional CPD topics and stimulating interactive and collaborative exploration.

The AMEE 2019 Annual Conference will bring a CPD track, two CPD Symposia and a myriad of posters and free communications on CPD.

We cordially invite you to join the AMEE CPD Community by applying to the CPD Special Interest Group (SIG) and by participating in the upcoming CPD events. CPD mirrors a harmonized diversity and scaffolds meaningful lifelong learning aiming at the best healthcare outcomes.

## Take Home Messages

• CPD is multiprofessional and entails a variety of perspectives from its multiple stakeholders
• Clinical and educational outcomes should both be patient centered
• CPD educators face brave new and challenging educational worlds ahead
• Good CPD research facilitates evidence available to inform and monitor clinical practice
• There should be a collective responsibility and leadership of all CPD stakeholders towards patient safety and quality improvement supported on effective CPD

## Notes On Contributors

Helena Filipe, MD, MSc-Medical Education, FSACME, AFAMEE is a Consultant of Ophthalmology at Hospital das Forças Armadas/PL-EMGFA and Hospital SAMS, Lisbon, Portugal. She currently serves the International Council of Ophthalmology (ICO) as chair of the CPD area of focus, serves the Association for Medical Education in Europe (AMEE) CPD Committee as co-chair, the Association for Research in Vision and Ophthalmology (ARVO) CME Committee, the Global Alliance for Medical Education (GAME) Education Committee and the Board of the Portuguese Medical Association College of Ophthalmology. She is an invited collaborator of the Department of Medical Education of the Faculty of Medicine of the University of Lisbon.

Dr. Samar Aboulsoud, MBBCH, MSc int. med, PhD, MSc Med Ed, FHEA, MAcadME A passionate medical educator who brings considerable experience in education with special interest in healthcare policy and change management with specific expertise in educational leadership, accreditation and regulation of healthcare systems. Dr Aboulsoud worked in several capacities in education, administration, quality and patient care in academic, corporate, government, publishing, and not-for-profit organizations. A nationally and internationally recognized leader in continuing medical education, with a deep interest in knowledge management and its effective translation to healthcare practice, with several committee and advisory board appointments and awards. She served as the Chief Executive Officer of the Qatar Council for Healthcare Practitioners. She was also the Director of Accreditation and Medical Education Department. In this position, she worked in collaboration with national and international partners in a number of national level projects towards establishing standards for medical education, accreditation as well as licensing of healthcare practitioners. She was the thought leader and project manager for the establishment of the national CME/CPD accreditation system for the state of Qatar.

## Declarations

Drs Samar Aboulsoud and Helena Prior Filipe are Guest Theme Editors for the theme of Continuing Professional Development in AMEE MedEdPublish.
