# Supplementary material for: Concluding Commentary: A Call for evidence in CPD and Lifelong Learning
Source: MedEdPublish (2016). 2019 Mar 26;8:70. [Version 1] doi: 10.15694/mep.2019.000070.1 (PMC10712605; doi:10.15694/mep.2019.000070.1)
Supplement: Peer review history

# Reviewer report: Reviewer response for version 1

**DOI:** 10.21956/mep.19802.r29793

**Published:** 2019-04-16

**Authors:** Trevor Gibbs

**Affiliations:** 1 AMEE

This review has been migrated. The reviewer awarded 5 stars out of 5 An excellent concluding commentary on a very important theme. To those of us who remember the early days of CME, this commentary shows us how far we have moved into the field of CPD. I enjoyed reading the paper and learned a lot, particularly enjoying how the papers demonstrated the variability of how we report our CPD activities.An essential read for all thos involved in the area - thank you

Reviewer Expertise:

NA

No decision status is available

# Reviewer report: Reviewer response for version 1

**DOI:** 10.21956/mep.19802.r29791

**Published:** 2019-04-15

**Authors:** Ken Masters

**Affiliations:** 1 Sultan Qaboos University

This review has been migrated. The reviewer awarded 5 stars out of 5 This is a really useful concluding commentary and synopsis of the papers submitted during this themed issue of CPD. The authors have analysed the papers in some detail, and have drawn out the most prominent strands of the 15 articles in the issue. The authors have also demonstrated that the papers show a healthy mixture of paper types, including “New education method or tool”, “Research articles”, “Practical tips and/or guidelines”, “Case study” and “Report of meeting or workshop”. They have further explored the paper types in some detail, addressing some of the more pertinent issues raised in those papers. Table 2 is a very useful summary of the papers, by type and keywords.Overall, a well-constructed and insightful summary of the theme’s contributions to our knowledge of CPD.

Reviewer Expertise:

NA

No decision status is available

# Reviewer report: Reviewer response for version 1

**DOI:** 10.21956/mep.19802.r29792

**Published:** 2019-03-28

**Authors:** Sateesh Babu Arja

**Affiliations:** 1 Avalon University School of Medicine

This review has been migrated. The reviewer awarded 4 stars out of 5 Once again I would like to congratulate the editors for taking up such an important topic in medical education 'Continuing Professional Development.' I liked the figure presented in this article representing the key stakeholders regarding CPD are patients, health care workforce, employers, regulators, societies, and policymakers. I liked the principles of CPD detailed in this closing editorial into four themes systematization, comprehensiveness, regulation, and accreditation. Regulation and accreditation play a significant role as some countries require revalidation or re-licensure or recertification or require certain credits whereas in some countries it is not mandatory. The other principle or theme I would like to add is outcomes. Any CPD activity being carried out should reflect in the form of outcomes. The authors have mentioned patient outcomes in this editorial. But even for academic faculty members, CPD activities should reflect the outcomes in the form of teaching methods and assessment methods employed or used by them in their academic work.

Reviewer Expertise:

NA

No decision status is available
